# Acute Myocardial Infarction Following *Androctonus crassicauda* Envenomation: A Case Report

**DOI:** 10.1155/cric/7231648

**Published:** 2026-06-19

**Authors:** Banafshe Nouri, Mohammad Fotouhi Siyah Pirani, Alireza Malekrah, Safa Mousavi

**Affiliations:** ^1^ Department of Cardiology, Mazandaran University of Medical Sciences, Sari, Mazandaran, Iran, mazums.ac.ir; ^2^ Department of Cardiology, Qazvin University of Medical Sciences, Qazvin, Qazvin, Iran, qums.ac.ir; ^3^ Department of Medicine, Tabriz University of Medical Sciences, Tabriz, East Azerbaijan, Iran, tbzmed.ac.ir

**Keywords:** acute myocardial infarction, *Androctonus crassicauda*, cardiotoxicity, heart failure, scorpion envenomation

## Abstract

**Background:**

Scorpion envenomation is a significant clinical problem in endemic regions. Cardiovascular complications are recognized, but acute myocardial infarction (AMI) following *Androctonus crassicauda* envenomation has not been previously reported.

**Case Presentation:**

A 20‐year‐old previously healthy male presented 2 h after a scorpion sting to the left ankle with severe chest pain, diaphoresis, and nausea. An ECG showed ST‐segment elevations in the anterior leads, and cardiac biomarkers were elevated. Echocardiography revealed severe hypokinesia of the left ventricular apex and septum (EF 30%). The patient received intravenous thrombolysis shortly after low‐dose polyvalent antivenom, with rapid improvement. Coronary angiography was not performed; thus, alternative mechanisms such as coronary vasospasm, toxin‐induced myocarditis, or Kounis syndrome cannot be excluded.

**Conclusion:**

This case highlights a rare but potentially life‐threatening cardiac complication of *A. crassicauda* envenomation. Clinicians in endemic regions should consider scorpion sting in the differential diagnosis of acute coronary syndromes in young patients without traditional cardiovascular risk factors. Early recognition, antivenom administration, and cardiovascular support are essential. Further studies are needed to clarify specific cardiotoxic mechanisms.

## 1. Case Presentation

A 20‐year‐old previously healthy male presented to the emergency department 2 h after a scorpion sting to the left ankle, complaining of severe, radiating chest pain, diaphoresis, and nausea. On initial examination, he was alert, with a blood pressure of 120/80 mmHg, heart rate of 110 bpm, and oxygen saturation of 98% on room air.

Electrocardiography revealed ST‐segment elevations in the anterior precordial leads (V1–V4). Cardiac biomarkers were markedly elevated, with troponin I rising to 2.5 ng/mL. Echocardiography demonstrated severe hypokinesia of the left ventricular apex and septum, with an estimated ejection fraction of 30%. Routine laboratory tests were significant for hyperglycemia (blood glucose 212 mg/dL) and mild leukocytosis.

Given persistent chest pain and dynamic ST‐segment changes, the patient received intravenous thrombolysis. A low dose of polyvalent scorpion antivenom was administered shortly before thrombolysis to neutralize circulating venom and mitigate sympathetic overstimulation. Rapid clinical improvement was observed, with resolution of chest pain, stabilization of vital signs, and gradual normalization of cardiac biomarkers.

Due to the markedly reduced ejection fraction, the patient was admitted to the intensive care unit for close hemodynamic and cardiac monitoring. Continuous assessment included blood pressure, heart rate, oxygen saturation, serial ECGs, cardiac biomarkers, and electrolytes. Supportive measures included oxygen therapy, intravenous fluids, analgesics, and cardiac medications as clinically indicated. No vasopressors or mechanical ventilation were required during hospitalization.

Follow‐up ECG on the next day showed T‐wave inversions in the precordial leads, consistent with evolving myocardial injury. Coronary angiography was recommended to rule out obstructive coronary artery disease; however, the patient declined and was discharged against medical advice, continuing outpatient follow‐up.

## 2. Discussion

Acute myocardial infarction following *Androctonus crassicauda* envenomation is rare, but the close temporal association between the sting and the onset of cardiac symptoms strongly suggests a causal relationship [1, 2]. Cardiac manifestations developed within 30 min, including chest pain, ST‐segment changes, and regional wall motion abnormalities on echocardiography, indicating a rapid physiological response to venom exposure [3].

The venom of *A. crassicauda* contains a complex mixture of neurotoxins and cardiotoxins capable of inducing myocardial injury through multiple mechanisms:

1. Coronary vasospasm: Neurotoxins trigger massive sympathetic activation, resulting in a catecholamine surge that can cause sudden coronary constriction, reducing myocardial perfusion [1, 4].
2. Toxin‐induced myocarditis: Direct effects on cardiomyocytes, including disruption of sodium and calcium channels, impair contractility and conduction, triggering inflammation and myocardial injury [3].
3. Kounis syndrome: Allergic reactions may lead to mast cell activation, histamine release, and coronary constriction, potentially precipitating ischemia [2].

Venom‐induced catecholamine and cortisol surges can also explain systemic findings such as hyperglycemia and leukocytosis, reflecting both stress response and immune activation [5, 6].

Management requires prompt intervention. In this patient, thrombolysis was administered for persistent chest pain, ST‐segment elevation, and elevated cardiac biomarkers. Polyvalent antivenom was given shortly before thrombolysis to neutralize circulating venom and reduce sympathetic overstimulation, potentially limiting cardiovascular toxicity [1, 2]. The timing of antivenom relative to thrombolysis was intended to optimize both myocardial reperfusion and early venom neutralization. Previous reports support the safety and efficacy of this combined approach, though data remain limited [3].

Given the markedly reduced left ventricular ejection fraction (~30%), the patient received intensive care with continuous hemodynamic monitoring, serial ECGs, cardiac biomarkers, and electrolytes. Supportive measures included oxygen therapy, intravenous fluids, analgesics, and cardiac medications as indicated. Close monitoring allowed timely detection of potential complications, including arrhythmias, cardiogenic shock, pulmonary edema, or recurrent ischemia [1, 2].

A limitation of this case is the absence of immediate coronary angiography, precluding definitive exclusion of underlying obstructive coronary disease. Therefore, alternative mechanisms—coronary vasospasm, toxin‐induced myocarditis, or Kounis syndrome—remain plausible [2]. Furthermore, literature on specific cardiotoxins in *A. crassicauda* venom highlights the need for further research into molecular mechanisms [4].

In summary, this case illustrates the integration of pathophysiological understanding, timely therapeutic intervention, and critical care management. Awareness of scorpion species, clinical manifestations, and optimized treatment strategies is crucial to improve outcomes in endemic regions [1, 2].

## Funding

No funding was received for this manuscript.

## Consent

Written informed consent for publication was obtained from the patient.

## Conflicts of Interest

The authors declare no conflicts of interest.

## Data Availability

The data that support the findings of this study are available from the corresponding author upon reasonable request.
